# Intention Attribution and the Development of Moral Evaluation

**DOI:** 10.3389/fpsyg.2018.02663

**Published:** 2019-01-07

**Authors:** Brooke C. Hilton, Valerie A. Kuhlmeier

**Affiliations:** Department of Psychology, Queen’s University, Kingston, ON, Canada

**Keywords:** moral development, moral reasoning, intention attribution, prosocial behavior, cognitive development, social development, infancy, child

## Abstract

Research with infants and toddlers suggests that even early in development, humans evaluate others by considering the outcome of an action in relation to the intention underlying it. When someone tries but fails to do a good deed, for example, it seems that it is “the thought that counts.” However, research with slightly older children in the preschool years has produced mixed results: in some cases, children are solely considering the positive or negative outcome of an action when evaluating others, while in others, intention attributions are integrated. Such contradictory findings have prompted debate about the development of moral reasoning. Here, we examine extant research on early moral evaluation and propose that differences in the way that task procedures present intention and outcome information can (1) support or preclude young children’s intention attribution and (2) alter the relative saliency or predominance of each kind of information. In turn, these differences would influence the frequency and degree to which young children generate intention-oriented moral evaluations.

## Introduction

The development of children’s moral evaluations of others has received considerable attention in psychology research. Of particular interest has been the question of when children acquire the ability to consider someone’s intentions when forming evaluations, instead of relying primarily on the positive or negative outcome of that person’s actions (e.g., *Is someone who accidently hurts someone else still blameworthy?*). The ability to integrate both intention and outcome information into one’s moral judgments is well established in adults (Cushman, 2008; Young and Saxe, 2009); however, seminal work on moral reasoning in children suggested that children’s moral judgments of others’ actions are outcome-based until approximately 8 years of age Piaget’s (1932/1965).

The “outcome-intention” shift posited by Piaget has been supported by studies showing that children make primarily outcome-based evaluations in the preschool years followed by intention-based evaluations later in childhood (e.g., Nelson, 1980; Zelazo et al., 1996; see Margoni and Surian, 2016, for a review). Yet, other findings have contradicted Piaget’s conclusions, suggesting that even very young children and infants do rely on intention information when evaluating social actors (e.g., Armsby, 1971; Behne et al., 2005; Dunfield and Kuhlmeier, 2010; Chernyak and Sobel, 2016; Woo et al., 2017). Given the findings, some have noted that the development of moral reasoning might be better characterized as a U-shaped curve (i.e., infants and older children incorporate intentions into their behavioral evaluations, though between these time points, preschool age children are outcome-based) rather than a linear shift from outcome to intention-based reasoning (e.g., Margoni and Surian, 2016). A remaining question is why this discontinuity – if there truly is one – exists. In this paper, we review extant research and examine whether differences in the way that moral reasoning tasks have been presented to children could influence the extent to which preschoolers show evidence of incorporating intention information into their moral evaluations.

## Weighting Intentions and Outcomes in Infancy and Early Childhood

Within the first 2 years of life, infants and toddlers show evidence of two foundational components of moral reasoning: an ability to differentiate intentional from accidental action and the ability to distinguish positively valenced social outcomes from negative ones. Woodward (1999), for example, demonstrated that 9-month-old and, to an extent, 5-month-old infants differentiate between goal-directed hand-grasps of an object and seemingly unintentional contact. By the age of 14 months, toddlers preferentially imitate actions that appear to be performed intentionally as opposed to those that have occurred by accident (Meltzoff, 1995; Carpenter et al., 1998; Olineck and Poulin-Dubois, 2005). In relation to valenced intentional actions, infants also distinguish between puppets that have helped versus harmed others (e.g., Premack and Premack, 1997; see Van de Vondervoort and Hamlin, 2017, for a review).

Furthermore, infants and toddlers appear to apply intentionality attributions to their evaluations of individuals who are engaging in positively- or negatively valenced actions. For example, 9-month-olds show more impatience toward someone unwilling to share than someone who is unable (Behne et al., 2005). By 13–16 months, infants anticipate that others will prefer an individual who has accidentally caused harm to one who has harmed intentionally (Choi and Luo, 2015) and prefer a failed helper to an intentional hinderer (Lee et al., 2015). In some contexts, infants themselves show preferential reaching to puppets who have intentionally provided help to another individual over those whose help was provided accidentally (Woo et al., 2017), and by 21 months, toddlers preferentially help another individual who was previously unable to provide them with a desired outcome (a toy) over one who was unwilling (Dunfield and Kuhlmeier, 2010). Taken together, research with infants and toddlers in the first 2 years of life would support the claim of an early-emerging ability to distinguish unintentional from intentional behavior that underlies social evaluation.

However, research regarding preschool children’s ability to evaluate others by integrating intention and outcome information has been mixed. Though young children often strongly weight outcome information in their evaluations (e.g., Helwig et al., 1995; Killen et al., 2011; Cushman et al., 2013), other research has found that young children consider intentions when choosing whom to act prosocially toward. By 3 years, children will selectively help someone who has intended, but failed, to help another over one who intended, but failed, to harm (Vaish et al., 2010), and children preferentially share with a puppet who has harmed them accidentally by knocking over their toys as opposed to one who did so on purpose (Chernyak and Sobel, 2016). As many of the tasks in which children have shown evidence of using intention information to guide their social judgments measured a non-verbal behavioral response, it has been hypothesized that observations of outcome-based evaluations among young children may occur as a result of difficulties with verbally reporting intention-based evaluations (e.g., Margoni and Surian, 2016). Yet, on some tasks that require verbal responses, children do show evidence of intention-based moral reasoning (e.g., Nelson, 1980; Yuill and Perner, 1988; Nobes et al., 2009). As such, it is likely that other factors have contributed to children’s poor performance on some moral evaluation tasks.

## Reconsidering the Variety of Intentional (And Unintentional) Behaviors

Recently, researchers have begun to reassess the way that intention attributions are discussed in the study of moral development. Some are critical of the focus that is put on the special status of *intentional* action, arguing that the true developmental accomplishment is children’s ability to determine when an action is *accidental* or *unintentional* (Rosset and Rottman, 2014). In support of this, Rosset and Rottman have drawn on past literature indicating that it is not a failure to consider intentional action, but rather a bias toward perceiving accidental actions as intentional that underlies children’s difficulties on moral evaluative tasks. They advocate for a re-imagining of the outcome-intent shift, instead putting the onus on the increasingly nuanced abilities of children to assess unintentional action.

Complementary to this approach, some researchers have argued that the current consideration of intentional and unintentional action – primarily in terms of intentional, attempted, and accidental action – is too simplistic, and they have advocated for a distinction to be drawn between two kinds of accidents (e.g., Shultz et al., 1986; Nobes et al., 2009; Woo et al., 2017). True accidents, they argue, in which outcomes are entirely beyond the possible control of the agent, differ from negligent accidents in which outcomes could have been prevented and may be due in part to actor carelessness. This distinction may help to explain the mixed results regarding the outcome-intention shift, as extant research typically has not separated truly accidental from negligent actors.

In a study that did vary the presentation of truly accidental and negligently accidental action, 10-month-olds preferentially reached for a puppet that had accidentally harmed another over one that did so intentionally, but did not show such a preference for a puppet that acted negligently (Woo et al., 2017). Nobes et al. (2009) similarly found that 3-year-olds rated negligent actors as if they had behaved intentionally when evaluating them on the basis of their accidental harms. These researchers suggest that while the ability to discriminate truly accidental from intentional action emerges early in development, negligent actors may be evaluated more similarly to intentional actors than to truly accidental ones.

Though the recent research provides valuable evidence, the call for researchers to distinguish preventable from unpreventable accidents when assessing children’s moral reasoning abilities is not new. Yuill and Perner (1988) found that when accidents were divided into those in which consequences were foreseeable and those in which consequences were unforeseeable, 3-year-olds rated unforeseeable accidents as being less blameworthy than intentionally harmful behavior, but only 6- and 7-year-old children made the same judgment about foreseeable harmful accidents. Additionally, Farnill (1974) found that preschool-aged children rate inept, or clumsy, harmers as more similar to intentional harmers in some contexts. Taken together, it appears that when study designs treat negligent and foreseeable accidents as if they were the same as unpreventable accidental action, we may underrepresent young children’s ability to incorporate intentionality information into their social evaluations.

## The Roles of Intention Attribution and Intention/Outcome Saliency in Children’s Moral Evaluations

The recent approaches described above recommend careful consideration of intentional and unintentional action in moral development research. We suggest that such considerations will also require researchers to examine whether intention attributions related to the to-be- evaluated behavior are likely to be made at all – and then utilized over and above the action’s outcome – given a child’s current level of cognitive ability and the available input. Specifically, in this section, we discuss the ways in which differences in how moral evaluation task paradigms present intention and outcome information could (1) support or preclude intention attribution and (2) alter the saliency of each kind of information. In turn, these differences would influence the extent to which young children generate intention-oriented moral evaluations.

As a first step in this proposal, we note that in research with infants and toddlers, to-be-evaluated behavior must be presented visually, without verbal storytelling, usually as an acted play or video depicting a set of events. These displays provide perceptual cues that can be used to infer the intention state underlying the action, including those that are likely highly familiar to infants such as verbal indications of intentional or accidental actions (e.g., the exclamation “there” or “whoops”), hand motions such as grasping or dropping, or emotion cues such as a surprised facial expression (e.g., Call and Tomasello, 1998; Carpenter et al., 1998; Olineck and Poulin-Dubois, 2005; Dunfield and Kuhlmeier, 2010). When intention (or lack thereof) can be read from the observed behavior rather than inferred as a hidden mental state, infants, toddlers, and young children may be more likely to incorporate intention attributions into their moral evaluations due to the close correspondence of the behavioral cues to the intention of the actor. Studies conducted in this way with preschool-aged children, find that even 3-year-olds use intention information to guide their subsequent social evaluations in these contexts (Call and Tomasello, 1998; Vaish et al., 2010, 2018; Chernyak and Sobel, 2016; see also Li and Tomasello, 2018).

The cognitive process underlying intention attribution in these cases is still not agreed upon, but at least two contenders can be described here. By one account, the experiences infants and toddlers have while acting in the world provide action-production information that leads to the development of a representational model of the actions (e.g., Sommerville and Woodward, 2005; Sommerville et al., 2005, 2008). Alternatively, the ability to recognize intentional action is not supported primarily by one’s own experience with action production but, instead, by a cognitive system that works with a rationality principle such that actions are seen as intentional if the motion path and end state fit the environmental constraints in an efficient, rational manner (e.g., Gergely et al., 1995; Csibra and Gergely, 1998; Csibra et al., 1999).

Regardless of the mechanism, we simply note that the cognitive load that is required in order to infer intention, or lack thereof, is reduced in these paradigms due to the close correspondence of external cues to the intention of the actor. In contrast, tasks that rely primarily on a verbal description of action (though commonly with inanimate props or still images) – which make up the majority of research done with young children (e.g., Armsby, 1971; Farnill, 1974; Imamoglu, 1975; Nelson, 1980; Yuill and Perner, 1988; Zelazo et al., 1996; Baird and Astington, 2004; Killen et al., 2011; Nobes et al., 2016; Ball et al., 2017) – often contain fewer behavioral cues indicating actor intention, potentially encouraging children to rely on the outcome information instead. Indeed, in some of the more complex scenarios used, an actor’s true positive or negative intention can only be determined if children are able to represent an actor’s mental state of knowledge or ignorance (e.g., an actor intentionally throws out a bag while cleaning a room, but she is unaware that it contains someone’s highly desired snack: Killen et al., 2011). Children do not reliably show adult-like intention-based moral evaluation with these scenarios until past age 5 years (Killen et al., 2011).

Even in instances in which knowledge state attributions are not required to determine an actor’s harmful or beneficent intention, verbally presented moral reasoning tasks are likely more cognitively demanding than visually presented tasks for young children. Working memory demands may be greater for verbally presented action, making the retention of a stable intention-state representation, relative to outcome information, difficult for young children. For example, 4-year-olds appeared to be operating at the limit of their ability in encoding whether a verbally presented action was performed accidentally or intentionally in Cushman et al. (2013). Consistent with this suggestion, adults under high cognitive load are more likely to misreport accidental actions as intentional (Buon et al., 2013).

Verbally presented moral evaluation tasks may also alter the salience of intention and outcome information due to the wording of the moral scenarios and test questions. For example, Nobes et al. (2016) demonstrated the effects of task wording in their replication of the Zelazo et al. (1996) study on the moral reasoning abilities of preschoolers. They found that when questions meant to elicit children’s moral evaluations of scenarios were repeated exactly as presented in the original study (e.g., *is it okay for Anne to hit an animal?*), the finding that young children’s evaluations are outcome-based was replicated. However, when the wording was altered such that it focused on properties of the agent (e.g., *Is Anne good, bad, or just ok?*) as opposed to properties of the action, children were more likely to include intention information in their moral judgments (e.g., Anne is good/ok in scenarios in which she accidently hit an animal). Additionally, Nelson (1980) found that whether intention information is presented before or after outcome information during the morally relevant story impacted whether preschoolers incorporated intention into their evaluations; 3-year-olds tended to base their moral judgments on the first negative cue presented in the test stories, regardless of whether this contained information about the actor’s intention or information about the action outcome. Thus, in some verbal tasks, children may view the first piece of morally relevant information as particularly salient and use it to anchor their moral evaluations.

Beyond task wording and the order of presented information, the importance of the relative saliency of intentions and outcomes can also be seen in studies that present attempted or accidental actions. Children tend to show comparatively early success on tasks that require them to evaluate an attempted-but-failed behavior (e.g., trying to share a toy but having it fall out of reach; Dunfield and Kuhlmeier, 2010), and Cushman et al. (2013) found that the outcome-intention shift in preschoolers’ moral judgments was greater for accidental actions than attempted ones (see also Baird and Astington, 2004; Margoni and Surian, 2017). Actor intentions may be relatively more salient in attempted action scenarios, in which there is no change in outcome (no newly positive or negative result), than in accidental ones, in which a new, unintended outcome is produced (e.g., Young and Saxe, 2009). Consistent with this claim, Young and Saxe (2009) found that intention-based moral evaluation of accidental but not attempted harm is correlated with activation in the right temporal-parietal junction in adults, which is implicated in intention reasoning. It is possible, thus, that accidental actions require a stronger, more robust representation of intention. The absence of changed outcomes in attempted actions may make it easier for young children to utilize intention attribution over outcome information.

In the present review, we have emphasized that intentionality attribution is necessary for intention-based moral evaluation, and when these attributions are within the conceptual abilities of young children, a discrete intention-outcome “shift” is not likely to be found. We also presented evidence that children (and in some cases, adults) may still rely on outcome information if this information is in some manner prioritized in the task scenario, even when intention attributions can or have been made. To be clear, our perspective does not imply that there is no moral development *per se* and only broader social and cognitive development (e.g., theory of mind, working memory); (e.g., Cushman, 2008; Cushman et al., 2013), have cogently argued against that conclusion, particularly in relation to decisions regarding punishment. Here, our focus has been on the integration of intention information within moral evaluative decision-making.

## Conclusion

Though it is true that children’s reliance on intention information across a wide variety of moral evaluative tasks becomes more robust over development, research with infants and young children suggests that evaluations of others can be intention-based, even early in development. Our early social-evaluative decision-making does not rely solely on the outcome of observed actions. However, the frequency or degree to which intentions are considered in evaluations is both paced by the child’s ability to construct an accurate representation of intention in the first place and encouraged (or discouraged) by the saliency of intention versus outcome information.

## Author Contributions

All authors contributed intellectually to the conceptualization of this perspective paper and read and approved the submitted version. The initial draft was written by BH.

## Conflict of Interest Statement

The authors declare that the research was conducted in the absence of any commercial or financial relationships that could be construed as a potential conflict of interest.
